# Phytochemical and Cytotoxic Investigations of *Alpinia mutica* Rhizomes 

**DOI:** 10.3390/molecules16010583

**Published:** 2011-01-14

**Authors:** Sri Nurestri Abdul Malek, Chung Weng Phang, Halijah Ibrahim, Norhanom Abdul Wahab, Kae Shin Sim

**Affiliations:** 1 Institute of Biological Sciences, Faculty of Science, University of Malaya, 50603 Kuala Lumpur, Malaysia; 2 Centre for Foundation Studies in Science, University of Malaya, 50603 Kuala Lumpur, Malaysia

**Keywords:** *Alpinia mutica*, cytotoxic activity, cancer cell line

## Abstract

The methanol and fractionated extracts (hexane, ethyl acetate and water) of *Alpinia mutica* (Zingiberaceae) rhizomes were investigated for their cytotoxic effect against six human carcinoma cell lines, namely KB, MCF7, A549, Caski, HCT116, HT29 and non-human fibroblast cell line (MRC 5) using an *in vitro* cytotoxicity assay. The ethyl acetate extract possessed high inhibitory effect against KB, MCF7 and Caski cells (IC_50_ values of 9.4, 19.7 and 19.8 µg/mL, respectively). Flavokawin B (**1**), 5,6-dehydrokawain (**2**), pinostrobin chalcone (**3**) and alpinetin (**4**), isolated from the active ethyl acetate extract were also evaluated for their cytotoxic activity. Of these, pinostrobin chalcone (**3**) and alpinetin (**4**) were isolated from this plant for the first time. Pinostrobin chalcone (**3**) displayed very remarkable cytotoxic activity against the tested human cancer cells, such as KB, MCF7 and Caski cells (IC_50_ values of 6.2, 7.3 and 7.7 µg/mL, respectively). This is the first report of the cytotoxic activity of *Alpinia mutica*.

## 1. Introduction

In the Zingiberaceae family, *Alpinia* is considered a large and taxonomically complex genus with 230 species widely distributed throughout tropical and subtropical regions of Asia [1]. Many *Alpinia* plants are medicinal herbs and have been reported to possess antioxidant, anti-inflammatory, anticancer, immunostimulating, hepatoprotective and antinociceptive activities [2]. *Alpinia mutica* is a rhizomatous, perennial and aromatic herb native to Peninsular Malaysia and Thailand. There are several variants of *Alpinia mutica* in the wild, but the type species are mainly distributed in the northern region of Peninsular Malaysia. The specimen used in this investigation was collected from Perak and closely resembled the type species. Traditionally this plant was used by the locals to treat flatulence and the fruits are used to reduce swelling. 

In 1996, a phytochemical investigation of *A. mutica* rhizomes by Sirat *et al*. [3], resulted in the isolation of four compounds, namely flavokawin B, pinocembrin, 5,6-dehydrokawain and 1,7-diphenyl-5-hydroxy-6-hepten-3-one. Additionally, a report by Mohamad *et al*. [4] reported that the dichloromethane extract of the *A. mutica* rhizomes displayed inhibitory activity towards lipid oxidation and possessed possible inhibitory effects on two Gram-positive microorganisms such as *Bacillus substilis* and methicillin-resistant *Staphylococcus aureus*. In 2004, Jantan *et al.* reported the isolation of alpinetin and 5,6-dehydrokawain from the fruits of *A. mutica* and the compounds showed inhibitory effects on PAF receptor binding to platelets. 

The present study aimed to investigate the cytotoxic activity of *A. mutica* rhizomes and identify the active ingredients that contribute to this activity. The experimental approach in the present study was based on bioassay-guided fractionation. In this endeavour, the crude methanol (MeOH) and fractionated extracts [hexane, ethyl acetate (EtOAc) and water (H_2_O)] of *A. mutica* rhizomes were firstly prepared for the preliminary cytotoxic activity assessment to identify the cytotoxic active extracts. The cytotoxic active extracts were subjected to further isolation and purification procedures to obtain the chemical constituents present in the extracts. Their structures have been established with the aid of extensive NMR spectroscopic and mass-spectral analyses. The identified chemical constituents were then further tested for their cytotoxic activity against the selected human cell lines. To our knowledge, this is the first report on the cytotoxic effects of *A. mutica*. 

## 2. Results and Discussion

### 2.1. Preliminary cytotoxicity screenings of the crude MeOH and fractionated extracts (hexane, EtOAc and H_2_O)

Table 1 shows the results of preliminary cytotoxicity screening of the crude MeOH and fractionated extracts (hexane, EtOAc and H_2_O) of *A. mutica* expressed as IC_50_ values, averaged from three experiments. According to the United States National Cancer Institute plant screening program, a plant extract is generally considered to have active cytotoxic effect if the IC_50_ value, following incubation between 48 to 72 h, is 20 μg/mL or less [6,7]. The crude MeOH extract was found to have no cytotoxic effect on all the human cancer and non-cancer cells tested (IC_50_ > 100 µg/mL in all cases). However, the fractionated extracts displayed better cytotoxic activity than the crude MeOH extract, as shown in Table 1. The hexane extract having IC_50_ values in the range of 31.6–58.7 µg/mL can be classified as possessing mild cytotoxic activity against the selected human cell lines, whilst the water extract did not show any cytotoxic activity against any of the human cell lines tested (IC_50_ > 100 µg/mL in all cases). The EtOAc extract was the only fraction that possessed high inhibitory effect against KB, MCF7 and CaSki cells (IC_50_ values of 9.4, 19.7 and 19.8 µg/mL, respectively) and moderate effect against HCT116 (IC_50_ values of 20.4 µg/mL). It can thus be concluded that the EtOAc extract was selectively toxic against the KB, MCF7 and CaSki cells, but not toxic against MRC-5 normal cells (IC_50_ values of 41.7 µg/mL). The active ingredients in the EtOAc extract may lead to some valuable compounds that may have the ability to kill cancer cells. Based on the above findings, a further chemical investigation was directed to the EtOAc fractionated extract of the crude MeOH extract. This led to the isolation of compounds **1-4**. 

**Table 1:** Cytotoxic activity (IC_50_ µg/mL) of crude MeOH and fractionated extracts (hexane, EtOAc and H_2_O) against human cancer and non-cancer cell lines.

Extracts	IC_50_ (µg/mL)						
	KB	MCF7	A549	CaSki	HCT116	HT29	MRC5
MeOH	>100	>100	>100	>100	>100	>100	>100
Hexane	31.6 ± 0.5	58.7 ± 3.4	41.2 ± 2.2	32.8 ± 0.3	36.1 ± 1.1	47.4 ± 1.6	41.2 ± 2.4
EtOAc	9.40 ± 0.5	19.7 ± 0.2	21.7 ± 1.7	19.8 ± 1.0	20.4 ± 3.2	24.2 ± 1.6	41.7 ± 0.3
H_2_O	>100	>100	>100	>100	>100	>100	>100
Doxorubicin^a^	0.27 ± 0.01	0.05 ± 0.01	0.58 ± 0.01	0.18 ± 0.06	0.24 ± 0.04	0.33 ± 0.03	0.40 ± 0.03

^a^ Doxorubicin was used as the reference compound.

### 2.2. Chemical characterization of compounds ***1-4***

**Figure 1:**
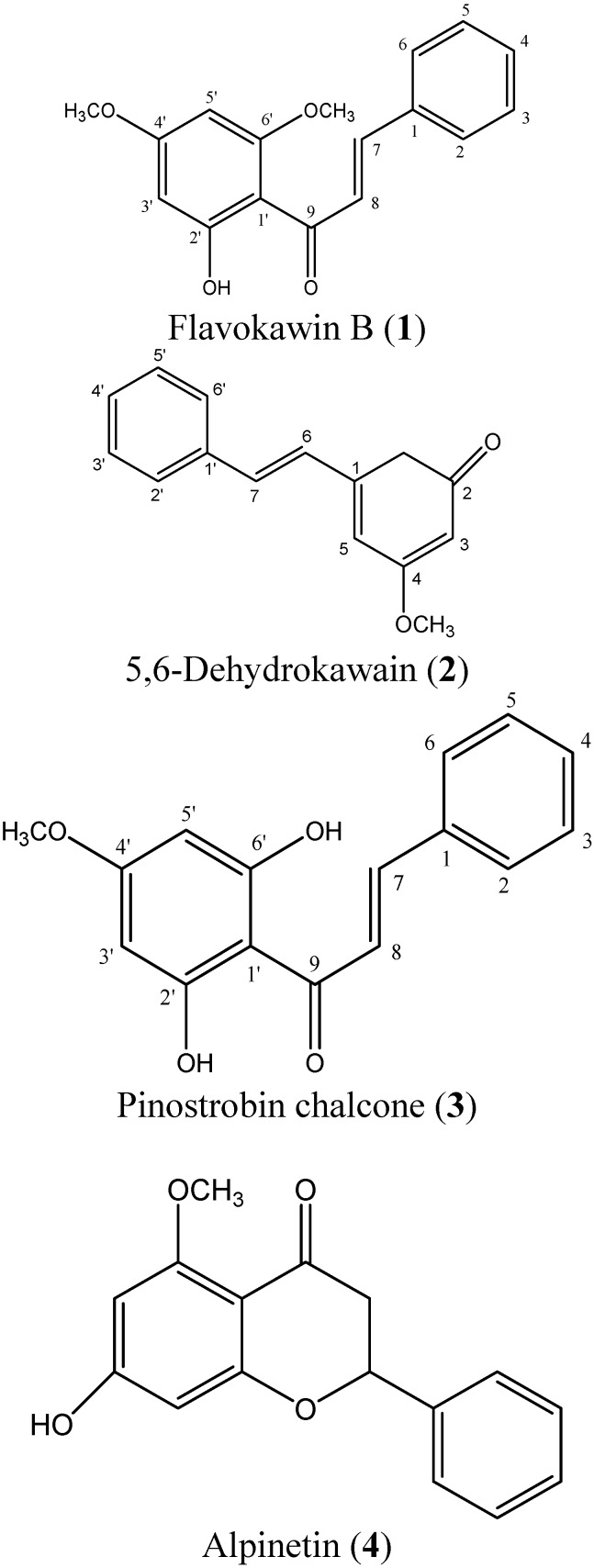
Structures of compounds **1**-**4**.

Compound **1** was identified as flavokawin B, **2** as 5,6-dehydrokawain, **3** as pinostrobin chalcone, and **4** as alpinetin. Compounds **1** and **2** have been reported for this plant [3], while compounds **3** and **4** were isolated from this plant for the first time. The structures of compounds **1**-**4** are illustrated in Figure 1. 

### 2.3. Cytotoxicity screenings of compounds ***1-4***

The compounds **1**-**4** were further tested against KB, MCF7, CaSki and HCT116 cells as the EtOAc extract showed high cytotoxic effect against KB, MCF7, CaSki cells and moderate effect against HCT116 cells (Table 1). The cytotoxicity screening results of of the compounds **1**-**4** are summarized in Table 2. According to the US NCI plant screening program, a pure compound is generally considered to have active cytotoxic effect if the IC_50_ value, following incubation between 48 to 72 h, is 4 μg/mL or less [6,7]. 

As shown in Table 2, pinostrobin chalcone (**3**) displayed remarkable cytotoxic activity against some of the tested human cancer cells, such as KB, MCF7 and CaSki cells (IC_50_ values of 6.2, 7.3 and 7.7 µg/mL, respectively) and also the normal MRC5 cells (IC_50_ value of 7.9 µg/mL). In addition, flavokawin B (**1**) in the EtOAc extract also contributed to the cytotoxic activity of EtOAc extract, as shown in Table 2. Flavokawin B (**1**) demonstrated strong activity against KB cells (IC_50_ 5.7 µg/mL) and HCT116 (IC_50_ 6.4 µg/mL). 5,6-Dehydrokawain (**2**) and alpinetin (**4**) did not show active cytotoxic activity against the selected cancer cell lines. 

Doxorubicin, which is widely used for the treatment of several human cancers, was used as the positive reference in the present study. Based on the result, it can be concluded that doxorubicin is not only cytotoxic against all the human cancer cell lines tested, but also exerts damage on the MRC5 non-cancer human cell line. Although the cytotoxicity of the compounds **1**-**4** are not as impressive as doxorubicin, they however have lower toxicity against the normal cells in comparison to this drug.

**Table 2:** Cytotoxic activity (IC_50_ µg/ml) of compounds **1**-**4** against human cancer and non-cancer cell lines.

Extracts	IC_50_ (µg/mL)				
	KB	MCF7	CaSki	HCT116	MRC5
Flavokawin B (**1**)	5.7 ± 0.4	49.3 ± 1.8	31.1 ± 1.3	6.4 ± 0.2	17.2 ± 0.5
5 ,6-Dehydrokawain (**2**)	20.7 ± 0.9	> 100	> 100	> 100	> 100
Pinostrobin chalcone (**3**)	6.2 ± 0.2	7.3 ± 0.6	7.7 ± 0.2	54.1 ± 4.6	7.9 ± 0.8
Alpinetin (**4**)	>100	> 100	>100	39.6 ± 2.0	> 100
Doxorubicin^a^	0.27 ± 0.01	0.05 ± 0.01	0.18 ± 0.06	0.24 ± 0.04	0.40 ± 0.03

^a^ Doxorubicin was used as the reference compound.

## 3. Experimental

### 3.1. General

TLC: Merck precoated plates (silica gel 60 F254) of 20.25 mm thickness. HPLC: Waters DeltaPrep system equipped with a Water PrepLC controller, quaternary pump, a vacuum degasser and UV detector (Waters 2487, Dual λ Absorbance Detector). The column used was a Chromolith Semiprep RP18 endcapped 100-10 mm. NMR spectra: JEOL 400 MHz FT NMR spectrometer at 400 MHz for ^1^H-NMR and at 100.40 MHz for ^13^C-NMR. Internal standard used in ^1^H-NMR and ^13^C-NMR spectra CDCl_3_ (δ: 77.0). GC-MS: Agilent Technologies 6980 N gas chromatography equipped with a 5979 Mass Selective Detector (70 eV direct inlet). The column used was HP-5ms (5% phenylmethyl- siloxane) capillary column (30.0 m × 25 mm × 25 µm) with helium as carrier gas at flow rate of 1 mL min^-1^. The column temperature was programmed initially at 100 °C, then increased to 300 °C and held for 10 minutes at 3 °C per minute. Absorbance value was measured by an ELISA reader (Molecular Devices EMax) to calculate the inhibition rate. Analytical grade solvents were purchased from Merck. 

### 3.2. Plant material

The rhizomes of *A. mutica* were collected from Perak, Malaysia. The samples were authenticated by Professor Dr. Halijah Ibrahim of Institute of Biological Sciences, Faculty of Science, University of Malaya, Malaysia and a voucher specimen (herbarium no. KLU 46177) was deposited at the herbarium of the Institute of Biological Sciences, Faculty of Science, University of Malaya, Kuala Lumpur, Malaysia.

### 3.3. Extraction and isolation for preliminary cytotoxic screening

The rhizomes of *A. mutica* were washed, dried and ground to fine powder (500.00 g). The powdered rhizomes were extracted with 80% MeOH (3 × 1.5 L) at room temperature yielding a MeOH extract (103.00 g, 20.60%). The MeOH extract (103.0 g) was further extracted with hexane to give a hexane-soluble extract (6.29 g, 6.10%) and a hexane insoluble residue. The hexane-insoluble residue was further partitioned between EtOAc–H_2_O (100 mL:100 mL) to give EtOAc-soluble extract (6.96 g, 6.75%) and a H_2_O extract (61.08 g, 59.30%). The crude MeOH and fractionated extracts (hexane, EtOAc and H_2_O) were dissolved in dimethyl sulfoxide (DMSO) with the exception of the H_2_O extract, to form stock solutions 20 mg/mL before testing. The final concentration of DMSO in test wells was not in excess of 1% (v/v).

### 3.4. In vitro cytotoxicity assay

The *in vitro* cytotoxic activity was studied by the neutral red cytotoxicity assay [5]. KB (epidermoid carcinoma cell line), MCF7 (hormone-dependent breast carcinoma cell line), A549 (lung carcinoma cell line), CaSki (cervical carcinoma cell line), HCT116 (colon carcinoma cell line), HT29 (colon carcinoma cell line) and non-human fibroblast cell line (MRC 5) were purchased from the American Tissue Culture Collection (ATCC). 

### 3.5. Extraction and isolation of pure compounds

The EtOAc extract of *A. mutica* (5.00 g) was subjected to column chromatography using a column packed with silica gel G_60_ (Merck). The column was eluted initially with chloroform (CHCl_3_) followed by CHCl_3_ enriched with increasing percentage of acetone (1%-40% acetone/CHCl_3_). Further elution was continued with 5% MeOH- CHCl_3_ and finally with 15% MeOH- CHCl_3_. One hundred mL fractions were collected. The eluents were then pooled to give six fractions (Fraction A–F) based on similarity of spots on TLC. The solvents were removed under reduced pressure in a rotary evaporator and the fractions were weighed. The isolation of the compounds was monitored by TLC using mixtures of CHCl_3_ and acetone. Fraction A was subjected to preparative TLC to give compound **1** (30.00 mg). Compound **2** (1.78 g) was isolated from fraction B from column chromatography by elution with 5% of Acetone: CHCl_3_. Elution with 20% acetone: CHCl_3_ gave fraction C which was further subjected to preparative HPLC to give compound **3** (7.50 mg). The mobile phase consisted of solvent A (acetonitrile) and B (0.1% trifluoroacetic acid in water) at gradient elution of 35% B for 0-3 minutes, 35%-40% B for 3-5 minutes, 40% B for 5-15 minutes, 40-85% B for 15-20 minutes and finally 85-35% B for 20-25 minutes. The flow rate was 5 mL/min, and UV detection was set at 220 nm and 254 nm. Compound **4** (5.00 mg) was recystallised from Fraction D using methanol. 

Flavokawin B (**1**) and 5,6-dehydrokawain (**2**) were identified through their mass spectral and NMR data which were consistent with published data [3,8]. Pinostrobin chalcone (**3**) was obtained as yellow needles; El-MS m/z (%): 270 (64), 269 (71), 253 (10), 193 (100), 167 (63), 103 (18), 77 (18), 55 (7). ^1^H-NMR (CDCl_3_, 400 MHz) δ: 3.88 (s, 3H, 2’-OCH_3_), 5.94 (d, 1H, *J* = 2.40 Hz, H-3’), 5.95 (d, 1H, *J* = 2.40 Hz, H-5’), 7.35 (m, 3H, *J* = 17.4 Hz, H3, H4, H5), 7.55 (dd, 2H, *J* = 2.40, 7.32 Hz, H2, H6), 7.65 (d, 1H, J=15.60, H9), 7.85 (d, 1H, *J* = 15.6 Hz, H7). ^13^C-NMR (CDCl_3_)δ: 55.39 (C-4’), 91.37 (C-5’), 95.96 (C-3’), 105.10 (C-1’), 127.74 (C-8), 128.21 (C-2,6), 128.86 (C-3,4,5), 135.53 (C-1), 141.95 (C-7), 163.42 (C-1’), 165.29 (C-4’), 167.30 (C-2’), 192.80 (C-9). Alpinetin (**4**) was identified through its mass spectral and NMR data which were consistent with published data [9,10]. 

## 4. Conclusions

In conclusion, the cytotoxic activities observed for *A. mutica* are ascribable to the active compounds flavokawin B (**1**) and pinostrobin chalcone (**3**). The cytotoxicity assay used in the present study could only provide important preliminary data to help select plant extracts or isolated compounds with potential anticancer properties. Further studies on the mechanism of cell death are needed to provide more convincing evidence. An investigation into this phenomenon is now underway. 
